# Rapid optimization of processes for the integrated purification of biopharmaceuticals

**DOI:** 10.1002/bit.27767

**Published:** 2021-05-04

**Authors:** Laura E. Crowell, Sergio A. Rodriguez, Kerry R. Love, Steven M. Cramer, J. Christopher Love

**Affiliations:** ^1^ Koch Institute for Integrative Cancer Research Massachusetts Institute of Technology Cambridge Massachusetts USA; ^2^ Department of Chemical Engineering Massachusetts Institute of Technology Cambridge Massachusetts USA; ^3^ Department of Biological Engineering Massachusetts Institute of Technology Cambridge Massachusetts USA; ^4^ Department of Chemical and Biological Engineering Rensselaer Polytechnic Institute Troy New York USA; ^5^ Center for Biotechnology and Interdisciplinary Studies Rensselaer Polytechnic Institute Troy New York USA

**Keywords:** biologics manufacturing, bridging buffer, high throughput process development, integrated purification, straight‐through chromatography

## Abstract

Straight‐through chromatography, wherein the eluate from one column passes directly onto another column without adjustment, is one strategy to integrate and intensify manufacturing processes for biologics. Development and optimization of such straight‐through chromatographic processes is a challenge, however. Conventional high‐throughput screening methods optimize each chromatographic step independently, with limited consideration for the connectivity of steps. Here, we demonstrate a method for the development and optimization of fully integrated, multi‐column processes for straight‐through purification. Selection of resins was performed using an in silico tool for the prediction of processes for straight‐through purification based on a one‐time characterization of host‐cell proteins combined with the chromatographic behavior of the product. A two‐step optimization was then conducted to determine the buffer conditions that maximized yield while minimizing process‐ and product‐related impurities. This optimization of buffer conditions included a series of range‐finding experiments on each individual column, similar to conventional screening, followed by the development of a statistical model for the fully integrated, multi‐column process using design of experiments. We used this methodology to develop and optimize integrated purification processes for a single‐domain antibody and a cytokine, obtaining yields of 88% and 86%, respectively, with process‐ and product‐related variants reduced to phase‐appropriate levels for nonclinical material.

## Graphical abstract

“Development and optimization of straight‐through chromatographic processes, wherein the eluate from one column passes directly onto another column without buffer exchange or adjustment of any kind, can be challenging. Conventional high‐throughput screening methods optimize each chromatographic step independently, with limited consideration for the connectivity of steps. Here, we demonstrate a method for the development and optimization of fully integrated, multi‐column processes for straight‐through purification.”

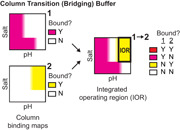

## 1 INTRODUCTION

Biopharmaceutical companies have increasingly diverse pipelines of recombinant protein‐based medicines including single‐domain antibodies, bi‐specific constructs, and enzyme replacements (Morrison, 2020). With the exception of monoclonal antibodies, however, most biologic products invoke unique manufacturing processes that require significant effort and time to develop. Regulatory pathways for accelerated approval, such as breakthrough designations, can also compress the time available to define a commercial strategy for manufacturing new products (Dye et al., 2016). Techniques to accelerate the development of new manufacturing processes for any protein therapeutic would facilitate the timely advancement of new potential treatments for patients.

Processes for purification are often particularly challenging to develop since they can involve three or more steps and there are a wide variety of chromatographic resins, operating modes, and operating conditions to test for a new product. High‐throughput screening has become a common method to develop and optimize processes for the purification of new products. These methods enable screening large numbers of resins and conditions in parallel using miniature columns or 96‐well plates (Łacki, 2012). The data from such screens often inform the optimal resin and operating conditions for an individual step in a multi‐stage sequence, but offer little consideration on the requirements for the connectivity of this step with other steps in the final process. This approach introduces additional operations to prepare intermediate eluates for the next steps (e.g., buffer exchanges, hold tanks, etc.) and these added steps can extend the total process, and cost, and introduce additional risks. Developing new techniques that consider the complete connectivity of steps in the final process could allow for more intensified processes by reducing unnecessary intermediate steps.

For monoclonal antibodies, there has been a growing interest in integrated and intensified manufacturing processes to reduce the time to operate and required space in facilities, since the steps and conditions for purifying similar molecules are relatively well understood (Garcia & Vandiver, 2017; Godawat et al., 2015; Steinebach et al., 2017; Warikoo et al., 2012). One recent area of research into integrated manufacturing is straight‐through chromatographic purification, where the eluate of one column is loaded directly onto the next column with minimal or no conditioning (pH or salt changes) (Hughson et al., 2017; Steinebach et al., 2017). Integrated, straight‐through chromatographic processes remove the need for hold tanks and additional unit operations, significantly reducing processing time, buffer usage, and space required in the manufacturing facility (Andersson et al., 2017; Löfgren et al., 2019).

It remains unclear, however, to what extent such integrated processes can be realized for other classes of molecules that vary in structure and complexity. Strategies for optimization, and design constraints applicable to any molecule, have been proposed for multi‐column integrated sequences with regard to sizing of columns and selection of flow rates (Andersson et al., 2017; Löfgren et al., 2019). These studies have relied on buffer exchanges between each chromatographic step, however, and thus have not considered the optimization of integrated straight‐through processes with minimal steps. New methodologies are needed for the design and optimization of processes for integrated, straight‐through purification of any protein therapeutic. Rapid development of such intensified purification processes could enable more agile manufacturing of a myriad of products on a single platform.

We have previously shown that a holistic approach to process development, coupled with a bench‐scale, integrated manufacturing platform, can reduce the time required to produce recombinant biologic products with phase‐appropriate quality attributes for clinical development (Crowell et al., 2018). Using this methodology, we can accelerate process development from sequence to purified product in as few as twelve weeks. This approach uses an in silico tool for the prediction of fully integrated, straight‐through purification processes based on a one‐time collection of host‐related data combined with conventional high‐throughput chromatographic screening data for each new target molecule (Timmick et al., 2018; Vecchiarello et al., 2019). Chromatographic sequences that remove both process‐ and product‐related variants can be predicted using this tool. While this method allows for rapid selection of resins for integrated processes, we reasoned a companion method for optimizing the buffer conditions used in the nominated process could further maximize yield and minimize impurities.

Here, we demonstrate a methodology to optimize the buffer conditions in integrated, straight‐through chromatographic processes with respect to yield and impurity removal using high‐throughput screening techniques. Resins were selected using our in silico tool for the prediction of fully integrated purification processes (Timmick et al., 2018). For the selected resins, potential operating ranges were identified through conventional single‐column screening with minimal analytics. We then carried out fully integrated (multi‐column) testing of the proposed operational area with more extensive analytics, including measurements of host‐cell protein, DNA, aggregate, and yield. Using these methods, we developed integrated processes for the purification of two different classes of proteins, a single‐domain antibody and a cytokine, with overall purification yields of 88% and 86% through two and three stages of chromatography, respectively, and process‐related impurities reduced below regulatory guidelines for nonclinical development.

## 2 MATERIALS AND METHODS

### 2.1 Protein production

Wild‐type *Komagataella phaffii* (NRRL Y‐11430) was modified to express G41, a single‐domain antibody, or G‐CSF, a cytokine, as described previously (Crowell et al., 2018). The biophysical characteristics of each molecule can be found in Table S1. Shake flask cultivations were conducted as described previously (Timmick et al., 2018), except rich defined media (Matthews, Kuo, et al., 2017) was substituted for complex media. 4% glycerol was added for outgrowth and 5% methanol/30 g/L sorbitol was added for production. 0.1% CHAPs (3‐[(3‐cholamidopropyl)dimethylammonio]‐1‐propanesulfonate hydrate) was added to the medium for G‐CSF cultivations. Additional supernatant was produced using the production module of the InSCyT system operated in perfusion mode (Crowell et al., 2018). In the bioreactor, temperature, pH, and dissolved oxygen were maintained at 25°C, 6.5, and 25%, respectively. All chemical reagents were purchased from Sigma‐Aldrich.

### 2.2 Resin selection

For G41, resins were selected based on our previously developed platform process for the purification of single‐domain antibodies (Crowell et al., 2021). This platform process was based on the purification processes predicted from our in silico tool for two different single‐domain antibodies (Timmick et al., 2018). The selected resins included CMM HyperCel and HyperCel STAR AX (Pall Corporation). For G‐CSF, our in silico tool was used exactly as described in Timmick et al., and the selected resins included CaptoMMC ImpRes, HyperCel STAR AX, and MEP HyperCel (GE Healthcare Life Sciences and Pall Corporation).

### 2.3 Determination of dynamic binding capacity

A full factorial design of experiment (DoE) was designed to model dynamic binding capacity using JMP® Pro 14.0.0. (SAS Institute Inc.). *K. phaffii* supernatant containing G41 was concentrated approximately 30‐fold using Amicon® Ultra‐15 Centrifugal Filter Units with 3 kDa membranes (MilliporeSigma). The concentrated supernatant was then diluted 15‐fold into the appropriate capture buffer. Nine experiments were conducted with capture buffers of 20 mM sodium citrate pH 4, 5, or 6 and conductivity 10, 20, or 30 mS/cm at all permutations. Conductivity was adjusted using sodium chloride. All experiments were conducted on a Tecan Freedom EVO® 150, controlled by EVOware Standard version 2.7.30.0 (Tecan Trading AG). The system was equipped with an eight‐channel liquid handling (LiHa) arm, an eccentric robot manipulator (RoMa) arm, 1 ml syringes, Te‐Shuttle, and Te‐Chrom modules. Absorbance was measured on an integrated Tecan Infinite M200 Pro using 96‐well UV transparent plates (Corning Inc.). Two hundred microliter prepacked OPUS® RoboColumns® were used (Repligen Corporation). Columns were equilibrated in capture buffer. The prepared supernatant was loaded up to 65 column volumes (CVs). The columns were then re‐equilibrated in capture buffer and eluted with 20 mM sodium phosphate, pH 8.0, 300 mM NaCl. Two hundred microliter fractions were collected during the load and elution steps and absorbance was measured at 280 nm and 260 nm. Absorbance measurements were corrected for liquid level using absorbance at A990‐A900 (Diederich & Hubbuch, 2017). The dynamic binding capacity was calculated as the amount of protein loaded onto the column when 20% breakthrough was reached. Protein concentration in the loaded supernatant was determined by size exclusion chromatography (SEC) (see below). JMP® Pro 14.0.0 was used to model the DoE (SAS Institute Inc.). Dynamic binding capacity for G‐CSF was modelled the same as above except that *K. phaffii* supernatant was spiked with purified G‐CSF to 0.3 mg/ml and loaded up to 100 CVs. Protein concentration in the loaded supernatant was verified by reversed‐phase liquid chromatography (RPLC). All chemical reagents were purchased from Sigma‐Aldrich.

### 2.4 Determination of potential operating regions

Binding maps for each product on the relevant resins were determined using linear‐gradient screens on the Tecan. Six hundred microliter OPUS® RoboColumns® were used (Repligen Corporation). A linear pH gradient was conducted at three salt concentrations (0, 150, and 300 mM sodium chloride). The pH gradients were performed using 12 buffers linearly varying the pH from pH A to B, produced using the Tecan Buffer Creation Wizard, version 1.2.0.0 (Tecan Trading AG). The gradients used for each column are as follows: CMM or MMC ImpRes (pH 5.8–8.0), STAR (pH 8.0–5.8), MEP (pH 8.0–3.6). All buffers above pH 5.7 were 20 mM sodium phosphate, and all buffers below pH 5.7 were 20 mM sodium citrate. Columns were equilibrated with capture buffer (20 mM sodium citrate, pH 5.0, 15 mS/cm for CMM and MMC ImpRes or 20 mM sodium phosphate, pH 8.5, 15 mS/cm for STAR and MEP) for 5 CVs, loaded with 10 (G41) or 20 (G‐CSF) CVs of product‐containing supernatant adjusted to pH 5.0 and 15 mS/cm for CMM and MMC ImpRes or to pH 8.0 and 15 mS/cm for STAR and MEP, re‐equilibrated with capture buffer for 5 CVs, eluted in the gradient for a total of 24 CVs, and stripped with high salt for 2 CVs. Two hundred microliter fractions were collected during the elution and salt strip and absorbance was measured as above. All chemical reagents were purchased from Sigma‐Aldrich.

The absorbance measurements were used to create chromatograms and then the chromatograms were used to create column binding maps. The protein was considered “not bound” at any conditions beyond the maximum absorbance peak of the respective chromatogram. The protein was considered “bound” at all conditions before the maximum absorbance peak for which the absorbance was less than 20% of the value at the peak maximum. The protein was considered “partially bound” at all conditions before the maximum absorbance peak for which the absorbance was greater than 20% of the value at the peak maximum. Column bridging maps were created by summing the CYMK color values for each contributing single‐column binding map.

### 2.5 Multi‐column design of experiments

JMP® Pro 14.0.0 was used to design the multi‐column DoE (SAS Institute Inc.). The Custom Design feature was used to design I‐optimal DoEs. Four continuous factors were modelled for G41 (capture pH, capture salt, bridging pH, and bridging salt) and six continuous factors were modelled for G‐CSF (capture pH, capture salt, bridging pH, bridging salt, elution pH, and elution salt). An additional blocking factor was added to represent which InSCyT system was used to perform the experiments. Blocking factors are typically added for variables that have an effect on the output, but are not of interest themselves. In this case, we expected the system used to have an effect because we know that the pumps on each system have slightly different flow rates (Table S2). Main effects, interaction and quadratic terms up to the second order were included in the model. Based on the DoE designs, 18 experiments were conducted for G41 and 30 experiments were conducted for G‐CSF (Tables S3 and S4). All experiments were executed on the InSCyT purification module (Crowell et al., 2018). All columns were equilibrated in the appropriate buffer before each run. Product‐containing supernatant was adjusted to match the conditions of the capture buffer using 100 mM citric acid and MilliQ water. For G41, the adjusted supernatant was loaded to 1 mg onto a 1 ml pre‐packed CMM HyperCel column (Pall Corporation), re‐equilibrated with capture buffer, washed with 20 mM sodium phosphate pH 5.8, and eluted with bridging buffer. Eluate from column 1 above 10 mAU was flowed through a 1 ml pre‐packed HyperCel STAR AX column (Pall Corporation). Flow‐through from column 2 above 10 mAU was collected. For G‐CSF, the adjusted supernatant was loaded to 1.6 mg onto a 1 ml pre‐packed MMC ImpRes column (GE Healthcare Life Sciences), re‐equilibrated with capture buffer, washed with 20 mM sodium phosphate, pH 5.8, and eluted with bridging buffer. Eluate from column 1 above 10 mAU was flowed through a 1 ml pre‐packed HyperCel STAR AX column (Pall Corporation). Flow‐through from column 2 above 10 mAU was loaded onto a 1 ml prepacked MEP HyperCel column (Pall Corporation), re‐equilibrated with bridging buffer, washed with 20 mM sodium citrate, pH 5.5, and eluted with elution buffer. Eluate from column 3 above 10 mAU was collected.

After data collection, JMP® Pro 14.0.0 was used to fit a model to the data (SAS Institute Inc.). Responses that did not show significant variation during the DoE experiments were excluded from the model, including aggregate for G41 and DNA for G‐CSF. All other responses were fit simultaneously. The fitting and analysis method (fitting personality) was set to standard least squares. Interaction and second‐order model terms were removed from the model sequentially, starting with the term with the highest *p* value, until all terms in the model had a *p* value below 0.1.

### 2.6 Analytical procedures

Samples were analyzed for host cell‐protein content using the *Pichia pastoris* 1st generation HCP ELISA kit from Cygnus Technologies according to the manufacturer's recommended protocol. Samples were analyzed for residual host‐cell DNA using the Quant‐iT dsDNA High‐Sensitivity Assay Kit (Invitrogen) according to the manufacturer's protocol except the standard curve was reduced to 0–20 ng. Unpurified samples were not analyzed for DNA content due to interference of media components with the Quant‐iT dsDNA High‐Sensitivity Assay Kit. Instead, typical DNA content of unpurified material produced in *K. phaffii* is used for comparison (Timmick et al., 2018). Sample concentration and aggregate percentage were determined on an Agilent 1260 HPLC system equipped with a diode array detector and controlled using OpenLab CDS software (Agilent Technologies). Aggregate was measured using an AdvanceBio SEC column (4.6 × 300 mm, 300 Å, 2.7 µm), with an AdvanceBio SEC guard column (4.6 × 50 mm, 300 Å, 2.7 µm) (Agilent Technologies). The column was operated at 0.25 ml/min and ambient temperature. The mobile phase was 150 mM sodium phosphate, pH 7.0 and total run time was 30 min. Sample injections volumes were 10 and 30 µl for G41 and G‐CSF, respectively. UV absorbance was collected at 214 and 280 nm. G41 concentration was determined using the same SEC method. G‐CSF concentration was determined using a PLRP‐S column (2.1 × 150 mm, 300 Å, 3 µm) operated at 0.5 ml/min and 60°C (Agilent Technologies). Buffer A was 0.1% (v/v) trifluoroacetic acid (TFA) (Thermo Fisher Scientific) in water and buffer B was 0.1% (v/v) TFA, 0.5% (v/v) water in acetonitrile (VWR International). A gradient of 45%–70% B was performed over 14 min; total method run time was 30 min. Sample injection volumes were 50 µl. Data analysis was completed using OpenLab CDS Data Analysis (Agilent Technologies).

## 3 RESULTS AND DISCUSSION

**Figure 1:**
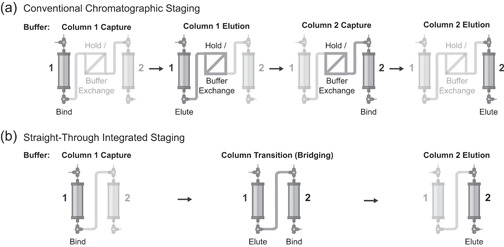
Schematic diagram of the key buffers required in a purification process comprised of two bind‐and‐elute steps using (a) conventional chromatographic staging or (b) straight‐through integrated staging

The key difference between conventional chromatographic processes and straight‐through processes is that some of the buffers used must interact with multiple columns in straight‐through processes by the very nature of their connectivity. Consider a conventional chromatographic process comprising two bind‐and‐elute steps: This process requires four key buffers—the capture and elution buffers for both columns 1 and 2 (ignoring the washing and cleaning buffers here for simplicity) (Figure 1a). Each of these buffers has a single purpose, and interacts with only one resin. For example, the column 1 elution buffer elutes the product from column 1. Because a buffer exchange occurs between the two columns, this buffer does not interact with column 2. In a straight‐through chromatographic process with two bind‐and‐elute steps, however, there are only three key buffers (Figure 1b). The elimination of a buffer exchange between columns 1 and 2 requires a new type of buffer that we define as the bridging buffer. The bridging buffer is used to transition the product from one column to another. This buffer facilitates two actions: eluting the product from column 1 and binding the product to column 2. While conditions for the column 1 capture buffer and the column 2 elution buffer could be identified and optimized using conventional, single‐column methods, new methods are required to identify and optimize the conditions for bridging buffers that consider their interaction with multiple columns.

**Figure 2:**
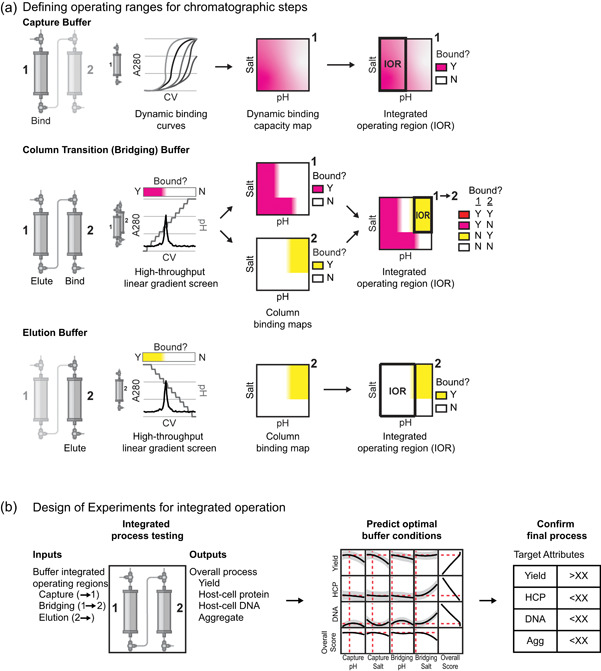
Schematic diagram of a two‐stage methodology for the optimization of buffer conditions for integrated, straight‐through purification processes to maximize yield and minimize impurities. (a) Potential integrated operating regions were determined for three main buffers in a two‐column purification process, the capture buffer, the column transition or bridging buffer, and the elution buffer. High‐throughput single‐column screens were used to create column binding maps. The binding maps show conditions where the product of interest is bound to the resin tested (pink: resin 1 or yellow: resin 2) and conditions where the product does not bind to the resin tested (white). Integrated operating regions were identified from the column binding maps. (b) DoEs was used to build a statistical model for the multi‐column process. The optimal buffer conditions to maximize yield and minimize impurities were predicted from the DoE model. CV, column volume; DoE, design of experiment [Color figure can be viewed at wileyonlinelibrary.com]

We aimed to develop an approach for the rapid optimization of buffer conditions used in integrated, straight‐through processes for the purification of biologics. Resins and initial operating conditions were selected using our in silico tool for the prediction of fully integrated purification processes; this tool allows for the prediction of resin sequences that will act in concert and orthogonally to recover the product while removing host‐cell proteins (Timmick et al., 2018). Importantly, an affinity capture step is not required for this tool, allowing this method for selecting resins to apply to any recombinant protein in principle. After selection of resins, buffer optimization was carried out in two stages (Figure 2). In the first stage, we conducted a set of range‐finding experiments on each individual resin to determine potential operating ranges for the capture, bridging, and elution buffers (Figure 2a). Potential operating ranges for the capture and elution buffers were then identified based on screens with single columns, while potential operating ranges for the bridging buffer were determined as the intersection of the applicable ranges for multiple columns. In the second stage of optimization, the identified operating ranges were used as inputs to build a statistical model for the multi‐column process, based on DoEs, to predict overall process yield and impurity levels, including host‐cell proteins, DNA, and aggregates (Figure 2b). The optimal buffer conditions were then identified using the statistical model to maximize yield and minimize impurities.

To test our approach, we sought to optimize the buffer conditions in the purification of a single‐domain antibody specific to influenza (G41), with respect to yield and removal of process‐ and product‐related impurities. Based on the platform purification process we had previously developed for single‐domain antibodies, we selected CMM HyperCel and HyperCel STAR AX as our resins for the purification of G41 (Crowell et al., 2021). We expected to run CMM in bind‐and‐elute mode followed by STAR in flow‐through mode.

**Figure 3:**
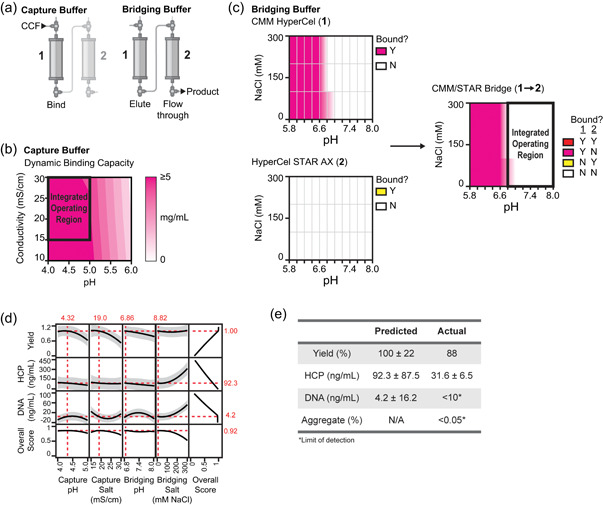
Optimization of buffer conditions in a two‐column, straight‐through purification process for a single‐domain antibody (G41). (a) Schematic of the two buffers optimized in the integrated purification of G41. (b) Response surface of dynamic binding capacity with respect to the pH and conductivity of the capture buffer. (c) Column binding maps for CMM HyperCel and HyperCel STAR AX. Conditions where G41 binds to the resin tested are pink (CMM) or yellow (STAR) and conditions where G41 does not bind to the resin tested are white. Column bridging map for the interaction between CMM and STAR resins. (d) Changes in the response variables (yield, HCP, and DNA) with changing buffer parameters. The 95% confidence interval is shown in gray. The overall score was calculated as in Equation (1), where each weight is equal to 0.33. Red lines indicate the conditions at which the overall score is maximized. Red numbers indicate the respective values at these optimal buffer conditions. (e) Yield, HCP, DNA, and aggregate values for the optimized G41 purification process. CCF, cell culture fluid; HCP, host‐cell protein [Color figure can be viewed at wileyonlinelibrary.com]

We aimed to optimize the capture buffer and the bridging buffer to maximize yield and minimize impurities in the integrated purification of G41 (Figure 3a). We identified appropriate capture buffer conditions for the first column (CMM) by conducting a full factorial DoE (9 experiments) to model dynamic binding capacity with respect to the pH and conductivity during product capture (Figure 3b). We began by performing a buffer exchange on clarified cell culture fluid (CCF) to remove media components that distort absorbance measurements at 280 nm, while maintaining host‐cell proteins in the CCF that may affect the binding of the protein of interest. Breakthrough curves were created for each capture condition to determine the dynamic binding capacity. We estimated the product to be about 61% of the total secreted protein mass in the CCF as determined by size‐exclusion chromatography. This is consistent with the range of 60%–80% purity reported for other recombinant proteins produced in *K. phaffii* cell culture (Matthews, Wright, et al., 2017). Due to this high initial purity, we assumed that changes in absorbance at 280 nm representing > 50% of the total observed change in absorbance were due to our protein of interest. Thus, absorbance was the only analytic required to track the protein of interest at this stage.

We defined the integrated operating region for the capture buffer as those conditions where > 75% of the maximum predicted binding capacity of the resin was reached. We also enforced a minimum load conductivity of 15 mS/cm because this condition represents a two‐fold dilution of our supernatant when recovered from a fermenter operating in perfusion mode. This limit could be adjusted for other fermentation methods, although we suggest avoiding dilutions larger than two‐fold. Greater dilutions would impede the benefits of integrated operation as larger dilution volumes would increase buffer usage, processing time, and manufacturing footprint. For G41, these requirements resulted in an acceptable operating region for the capture buffer in the range of pH 4.0–5.0 and conductivity 15–30 mS/cm (Figure 3b).

We next determined the potential operating conditions for the bridging buffer, the buffer used to transition the product from column one to column two. Screens of linear gradients varying pH at three salt concentrations were performed to identify the binding characteristics of the product with each resin. Again, due to the high initial purity from our host organism (60–80%), offline measurements of absorbance (280 nm) were sufficient as the only analytic to track our protein of interest at this stage. In this case, the peak with the maximum area was considered the product‐containing peak. Binding maps were constructed for each resin, indicating the conditions at which the product is bound or not bound (Figure 3c). Summing the binding maps for each column revealed potential operating conditions for a bridging buffer to enable integrated purification (Figure 3c). Since we intended to run CMM in bind‐and‐elute mode followed by STAR in flow‐through mode, we sought conditions for the bridging buffer where the product does not bind to either resin (white). We selected the acceptable operating range for the bridging buffer as pH 6.8–8.0 and conductivity 0–300 mM NaCl (Figure 3c). (To keep the DoE design simple, the range of each input should be independent of other inputs. In other words, a rectangular design space will require fewer experiments than a more complicated design space. We therefore chose not to include pH 6.6, 150–300 mM salt in the integrated operating region.) With the second column operating in flow‐through mode, we did not need to explore final elution conditions for this molecule.

In the second stage of optimization, an I‐optimal DoE was designed, varying capture pH, capture conductivity, bridging pH, and bridging conductivity of the fully integrated (two‐column) process. In an I‐optimal DoE, the prediction variance over the entire design space is minimized. In this case, response prediction was more important than estimating parameters. This type of DoE is typically good for predicting responses, determining optimum operating conditions, and determining regions in the design space where the response falls within an acceptable range (Goos & Jones, 2011).

The boundaries of the DoE were set using the potential operating conditions determined in the first stage of optimization. Based on the DoE, eighteen fully integrated purifications were carried out on our custom‐built InSCyT system (Crowell et al., 2018). The resulting purified samples were then analyzed for yield, host‐cell protein, host‐cell DNA, and aggregate. The measured aggregate was below the level of detection in our assays for all experiments. Product yields ranged from 1.9% to 99.7%, host‐cell protein concentrations ranged from 25 to 412 ng/ml, and DNA concentrations ranged from <10 to 107 ng/ml (Table S5).

A statistical model was then built using these data to predict yield, HCP and DNA based on capture pH, capture conductivity, bridging pH, and bridging conductivity (yield *R* ^2^_adj_ = 0.76, HCP *R* ^2^_adj_ = 0.80, DNA *R* ^2^_adj_ = 0.81). Residuals for each response with respect to each input variable were randomly distributed, indicating an acceptable model fit (Figure S1). We found that capture pH and capture salt had significant effects on yield, with increasing pH or salt leading to decreased yield (Figures 3d and S2). The conductivity of the bridging buffer had significant effects on both host‐cell protein and host‐cell DNA, with higher bridging salt leading to higher impurity levels (Figures 3d and S2). Interestingly, interactions between capture conditions and bridging conditions had significant effects on both yield and DNA (Figure S2). In traditional single‐column optimization, these interactions may have been missed during initial development of each column.

An overall score was calculated to select a single set of optimal buffer conditions (Equation 1). In this case, the overall score maximizes yield, minimizes host‐cell proteins, and minimizes DNA. *W*_X_ represents the weight of each factor and the sum of all weightings should be equal to one. Here, each weight was set to 0.33 so that all criteria were equally weighted because, at this stage, we were equally interested in maximizing yield and minimizing host‐cell protein and DNA. We predicted the optimal conditions for each buffer by maximizing the overall score (Figure 3d). The predicted optimal buffer conditions used low pH and salt in the bridging buffer (6.8 and 10 mM NaCl, respectively) to minimize HCP and DNA. The capture conditions were pH 4.3 and 20 mS/cm:

(1) $$\begin{array}{l} \begin{array}{c} \mathrm{Overall}\;\mathrm{Score}=\exp[W_{\mathrm{Yield}}*\log\left(\frac{\mathrm{Yield}}{{\mathrm{Yield}}_{\max}}\right)\\ \;+W_{\mathrm{HCP}}*\log\left(1-\frac{\mathrm{HCP}}{{\mathrm{HCP}}_{\max}}\right)\\ \;+W_{\mathrm{DNA}}*\log\left(1-\frac{\mathrm{DNA}}{{\mathrm{DNA}}_{\max}}\right)] \end{array} \end{array}$$

We conducted a purification with the predicted optimal buffer parameters and achieved a yield of 88%, host‐cell protein concentration of ~31 ng/ml (172 PPM), and DNA and aggregate levels below our level of detection. These results were consistent with our model predictions (Figure 3e). Compared to the original purification process developed for this molecule (Crowell et al., 2021), the optimized process improved yield from ~15% to 88%, while maintaining process‐related impurity removal.

To test the effect of load challenge on our optimal process, we ran a purification with the same buffer conditions and a higher load challenge. Based on the dynamic binding capacity model built previously (Figure 3b), we predicted that the binding capacity at our optimal capture conditions would be approximately 6 mg/ml. We therefore executed a purification using our optimal conditions and a load challenge of approximately 6 mg/ml. We recovered 100%* of the product with HCP at ~43 ng/ml (183 PPM) and DNA and aggregate below the level of detection (*measured recovery was 111%).

We next sought to test our methodology with a more complicated three‐stage integrated purification. We selected G‐CSF, a clinically relevant molecule for which we had previously developed a three‐step integrated purification process (Crowell et al., 2018; Timmick et al., 2018). Our initial purification process utilized Capto MMC ImpRes as a capture column operated in bind‐and‐elute mode, followed by HyperCel STAR AX in flow‐through mode, and finally MEP HyperCel in bind‐and‐elute mode. While this initial process had acceptable overall yield (~80%) and impurity removal, we hypothesized the process could be optimized further to increase yield while maintaining impurity removal by adjusting the buffer conditions.

**Figure 4:**
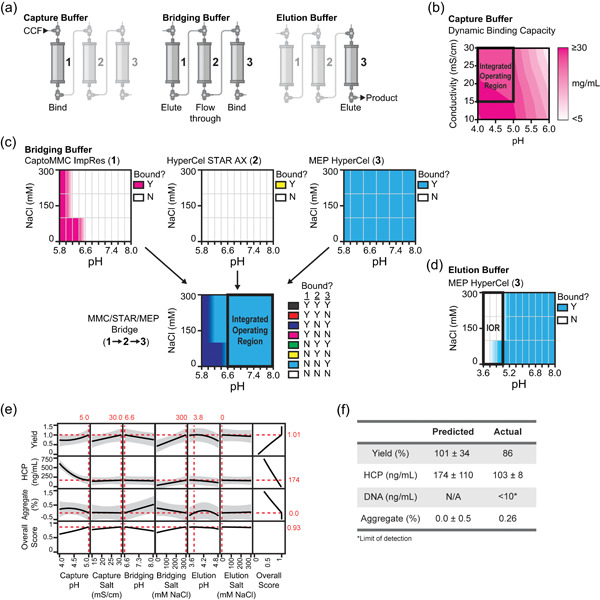
Optimization of buffer conditions in a three‐column, straight‐through purification process for a G‐CSF. (a) Schematic of the three buffers optimized in the integrated purification of G‐CSF. (b) Response surface of dynamic binding capacity with respect to the pH and conductivity of the capture buffer. (c) Binding maps for CaptoMMC ImpRes, HyperCel STAR AX, and MEP HyperCel. Conditions where G‐CSF binds to the resin tested are pink (CaptoMMC ImpRes), yellow (STAR), or blue (MEP) and conditions where G‐CSF does not bind to the resin tested are white. Column bridging map for the interaction between the CaptoMMC ImpRes, STAR, and MEP resins. (d) Column binding map for the final resin, MEP HyperCel. (e) Changes in the response variables (yield, host‐cell protein, and aggregate) with changing buffer parameters. The 95% confidence interval is shown in gray. The overall score was calculated as in Equation (1), where each weight is equal to 0.33. Red lines indicate the conditions at which the overall score is maximized. Red numbers indicate the respective values at these optimal buffer conditions. (f) Yield, HCP, DNA, and aggregate values for the optimized G‐CSF purification process. CCF, cell culture fluid; HCP, host‐cell protein [Color figure can be viewed at wileyonlinelibrary.com]

We aimed to optimize the capture buffer, the bridging buffer, and the elution buffer to maximize yield and minimize impurities in the integrated purification of G‐CSF (Figure 4a). We identified appropriate capture buffer conditions for the first column by conducting a full factorial DoE (9 experiments) to model dynamic binding capacity as described above (Figure 4b). Similar to with G41, we enforced minimum load conductivity of 15 mS/cm in the selection of the integrated operating region to minimize the required dilution from cell culture supernatant. We next determined the optimal conditions for the bridging buffer. Due to the modes of operation for each resin in this process (bind‐and‐elute → flow‐through → bind‐and‐elute), the bridging buffer must interact with all three resins. That is, the bridging buffer must allow the product to elute from the first column, flow‐through the second column, and bind to the third column (Figure 4a). We therefore created binding maps for all three resins, as described above, and summed all three maps to determine the potential operating range for the bridging buffer (Figure 4c). Here, we sought conditions where the product does not bind to either of the first two resins, but does bind to the third resin (light blue). Finally, the binding map created for the third resin, MEP HyperCel, was used to identify potential operating conditions for the elution buffer (Figure 4d).

An I‐optimal DoE was built by varying capture pH, capture conductivity, bridging pH, bridging conductivity, elution pH, and elution conductivity. The impact of these conditions on process yield, HCP, and aggregate were modelled (yield *R* ^2^_adj_ = 0.60, HCP *R* ^2^_adj_ = 0.77, aggregate *R* ^2^_adj_ = 0.26). Residuals for each response with respect to each input variable were randomly distributed, indicating an acceptable model fit (Figure S3). The measured DNA concentration was below the level of detection in our assays for all experiments. Product yields ranged from 0.0% to 86.3%, host‐cell protein concentrations ranged from 18 to 698 ng/ml, and aggregate ranged from <0.05% to 1.6% (Table S6). Missing data (impurity levels for samples with 0% recovery) were imputed for each response variable using multivariate normal imputation (Schäfer & Strimmer, 2005).

The conductivity of the bridging buffer and pH of the elution buffer had significant effects on yield, with higher bridging salt and lower elution pH increasing yield (Figures 4e and S4). Increased salt in the capture or bridging buffer led to higher host‐cell protein levels while increased pH in the capture or elution buffer decreased host‐cell protein levels (Figures 4e and S4). Few variables were found to be significant in predicting aggregate levels and the adjusted R‐squared score for this response showed that our model fit was poor (Figures 4e and S4). This result may be because the range of measured aggregate in these experiments (<0.05–1.6%) is too low to distinguish significant effects from measurement noise. As expected, the system flowrate (*F*) had a significant effect on predicting yield and residual host‐cell proteins (see Section 2; Figure S4).

As with G41, an overall score was defined to select a single set of optimal buffer conditions. In this case, yield was maximized, and host‐cell protein and aggregate were minimized, with each criteria weighted equally (Equation 1). The predicted optimal buffer conditions included high capture pH to minimize host‐cell protein, low bridging pH to minimize aggregate, and high bridging salt and low elution pH to maximize yield (Figure 4e).

The predicted optimal process for G‐CSF was executed and achieved a yield of 86%, HCP at 102.6 ng/ml (650 PPM), 0.26% aggregate, and DNA below our limit of detection (Figure 4f). These results were consistent with our model predictions. While yield increased and percent of aggregate decreased compared to the original process, residual host‐cell proteins also increased (Timmick et al., 2018). We note that the host‐cell protein challenge in the purifications presented here was higher than those used in the evaluation of the original process (Table S7). We hypothesize that this higher host‐cell protein challenge results from the fermentation, as the material used in this study came from a bioreactor while the material used in the previous study came from a shake flask. If the host‐cell protein levels obtained by this “optimal” process were deemed too high, however, the weighting of each factor in the overall score could be adjusted such that the minimization of host‐cell proteins was given more importance. This would not require any additional experiments other than testing of the new optimal conditions.

The confidence intervals on the model predictions for G‐CSF are fairly large, with 33% and 63% variance for yield and host cell protein concentration, respectively (Figure 4e,f). This variance is likely due to the fact that some of the experiments in the DoE resulted in 0% recovery. Impurity data could not be collected in those cases, and was imputed using multivariate normal imputation, weakening the predictive abilities of the statistical model. All experiments resulting in 0% recovery used the bridging buffer at pH 6.6 and 0 mM NaCl. Based on in‐process UV data, we determined that the product did not elute from the capture column at this condition, contrary to what was predicted in the binding screens. This outcome may be due to the different experimental platforms used to collect the binding data and to conduct the DoE experiments. One potential mitigation for such differences may be strict selection of potential operating regions in the first stage of optimization in future experiments. For example, using the range of pH 6.8–8.0 instead of pH 6.6–8.0 for the bridging buffer likely would have prevented this variance. Furthermore, conditions along the edge of the integrated operating region (pH 6.6 in this case) will likely not be robust. Nonetheless, using this two‐stage optimization methodology for the selection of buffer conditions, we realized a process for the integrated purification of G‐CSF with high yield and acceptable impurity removal.

## 4 CONCLUSIONS

We have presented a methodology for optimizing the buffer conditions used in integrated, straight‐through chromatographic processes, including conditions for the bridging buffer, utilizing high‐throughput screening technologies and design of experiments. Here, we have demonstrated this optimization on both two‐column and three‐column integrated purification processes, obtaining yields of 88% and 86%, respectively, with process‐ and product‐related variants below typical values for advancing nonclinical development (1000 PPM for HCPs and 10 ng/dose for DNA) (Jawa et al., 2016; The European Agency for the Evaluation of Medicinal Products, 1997; World Health Organization, 2013). This method is the only optimization strategy to our knowledge that specifically optimizes buffer conditions for straight‐through chromatographic processing, removing the need for additional intermediate steps such as hold tanks and buffer exchanges. The approach for optimizing buffers described here can be combined with a method we previously reported for selecting resins compatible with integrated purification (Timmick et al., 2018), along with strategies for optimization of column sizing and flow rates (Andersson et al., 2017; Löfgren et al., 2019), to enable the holistic development of integrated purification processes for non‐platform molecules.

Importantly, due to the high initial product purity obtained from secreted expression of proteins using *K. phaffii*, all of the initial range‐finding experiments in the first stage of the optimization were conducted using only cell culture supernatant and did not require any pre‐purification or product‐specific analytics. The simplicity of this expression host allows our methods to be used for a wide variety of products early in their development cycle. For the second stage of development (the DoE studies), product‐specific metrics such as SEC and/or RPLC were developed to measure yield and aggregate. Conditions identified in the first stage of optimization can be used to recover the product in a partially purified form to enable the development of product‐specific assays for tracking product‐related variants. This feature may be particularly useful for products which do not interact with any common affinity resins, as initial purification for such products is typically a challenge.

The optimization strategy here considers the entire purification sequence as a single unit operation, as opposed to each column being its own procedural step. The DoE‐like investigation of the multi‐column purification can follow a quality‐by‐design (QbD) approach, examining the relationship between critical process parameters (buffer conditions at each step) and critical quality attributes (host‐cell proteins, DNA, and aggregate). Furthermore, the criteria for optimality can be adjusted to fit product specific target product profiles (TPPs). For example, yield requirements may be less important during the early stages of preclinical development, where only small amounts of protein are needed to test the safety and efficacy of a candidate product. In this case, yield could be weighted lower in the optimal score to reflect these constraints. Additional improvements to the optimization equation could include acceptable targeted limits for impurity levels (such as < 100 PPM HCP). The holistic methods presented here could also be used to establish a single design space across the entire purification process, rather than separate design spaces for each purification step. As stated in ICH Q8, a design space that spans multiple unit operations can be used in filing and may provide more operational flexibility as compared to single unit operation design spaces (U.S. Department of Health and Human Services Food and Drug Administration Center for Drug Evaluation and Research & Center for Biologics Evaluation and Research, 2009).

An additional benefit of the methods presented here is a reduction in the total number of experiments required to optimize the purification buffers. In the case of G41, our DoE required 18 experiments, whereas a DoE on each individual column would have required 27 experiments (18 on the capture column and 9 on the flow‐through column). In the case of G‐CSF, our methods required 30 experiments, compared to the 45 experiments required to optimize each column individually ((18 for the capture column, 9 for the flow‐through column, and another 18 for the final polish column). In both instances, our methods represent a 33% reduction in number of experiments. This reduction in number of experiments is partially due to the reduced number of buffers used in straight‐through chromatography, and presents an additional potential advantage on top of reduction in buffer usage, processing time and manufacturing footprint.

While the methods presented here focus on multi‐column, straight‐through chromatography, we envision that the two‐step optimization approach of identifying potential operating regions followed by integrated modelling of a multi‐step process could be applied to the optimization of any integrated unit operations. Other examples could include production and formulation operations as well as alternative purification operations such as membrane chromatography or precipitation. The integrated modelling could invoke DoE as presented here, or mechanistic or hybrid models as they are available. Mechanistic or hybrid models may have increased predictability across multiple molecules. We believe that techniques for the rapid optimization of integrated processes such as those presented here could speed the development of integrated and continuous processes for new products and potentially speed the translation from sequence to first‐in‐human studies for novel biologics and vaccines.

## CONFLICT OF INTERESTS

Laura E. Crowell, Kerry R. Love, Steven M. Cramer, and J. Christopher Love have filed patents related to the InSCyT system and methods. Kerry R. Love and J. Christopher Love are co‐founders and consultants to Sunflower Therapeutics PBC.

## AUTHOR CONTRIBUTIONS

Laura E. Crowell, Kerry R. Love, Steven M. Cramer, and J. Christopher Love conceived and designed experiments. Laura E. Crowell and Sergio A. Rodriguez performed purification experiments, quality assessments, and data analysis. Laura E. Crowell, Kerry R. Love, Steven M. Cramer, and J. Christopher Love wrote the manuscript. J. Christopher Love, Steven M. Cramer, and Kerry R. Love designed the experimental strategy and supervised analysis. All authors reviewed the manuscript.

## Supporting information

Supporting information.

## Data Availability

The datasets generated and analyzed in this study are available from the corresponding author upon reasonable request.
