# Supplementary material for: Rapid optimization of processes for the integrated purification of biopharmaceuticals
Source: Biotechnol Bioeng. 2021 May 4;118(9):3435–46. doi: 10.1002/bit.27767 (PMC8453909; doi:10.1002/bit.27767)
Supplement: Supplementary file 1 — Supporting information. [file BIT-118-3435-s001.pdf]

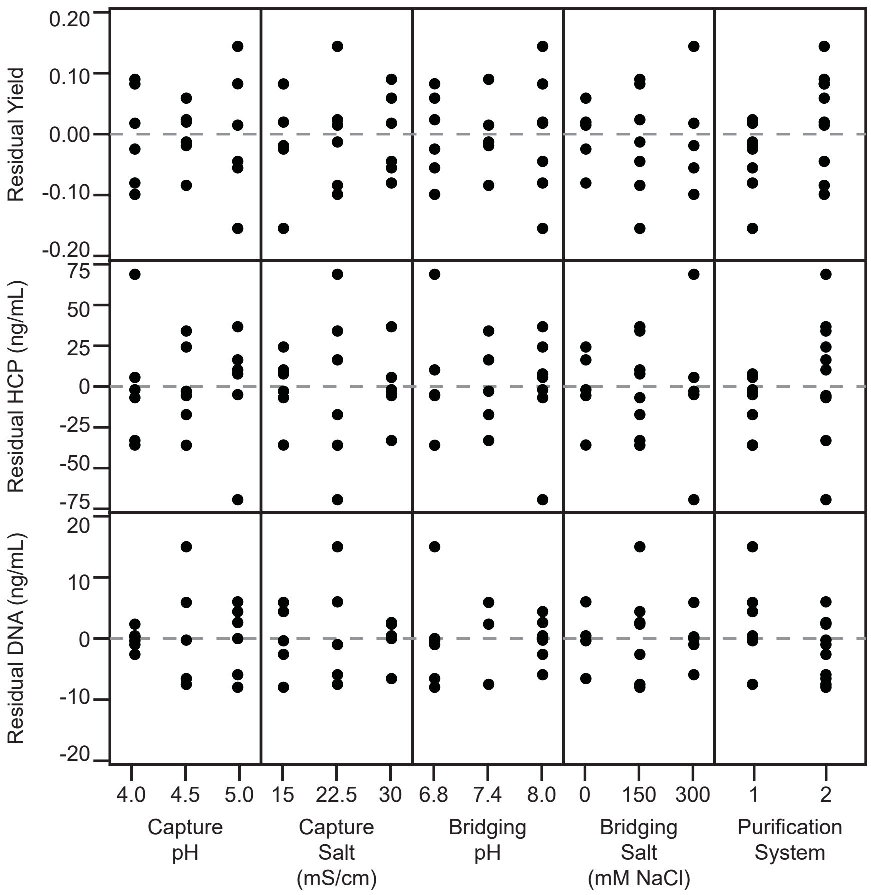

Supplementary Figure 1. Residual plots for the G41 process model. Residuals are shown for each response (yield, host-cell protein (HCP), and DNA) with respect to each input variable (capture pH, capture salt, bridging pH, bridging salt, and purification system).

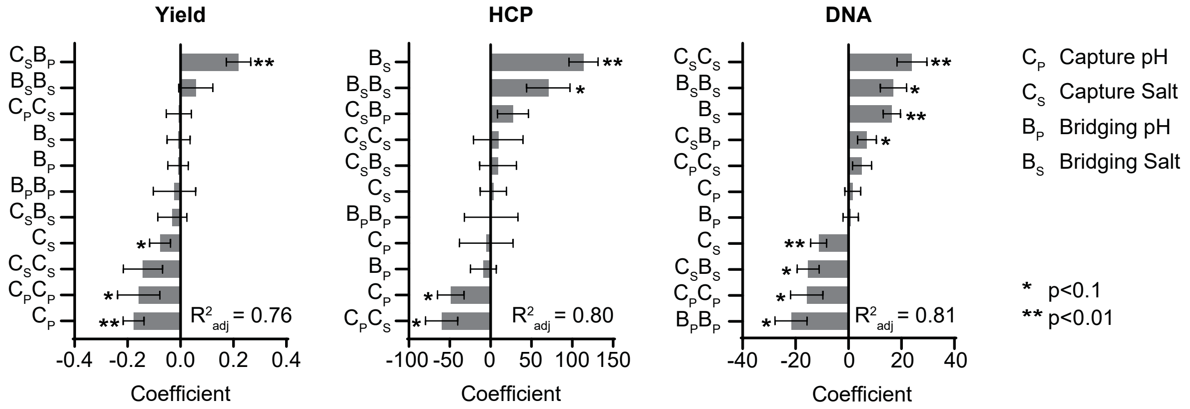

Supplementary Figure 2. Coefficient estimates for each G41 process model parameter with respect to each response variable including yield, host-cell protein (HCP), and DNA. Coefficients significantly different than zero based on a two-tailed t-test are marked with an asterisk. Error bars represent standard error. The adjusted R-squared score is shown for each response. C_p – capture buffer pH; C_s capture buffer salt; B_p – bridging buffer pH; B_s – bridging buffer salt

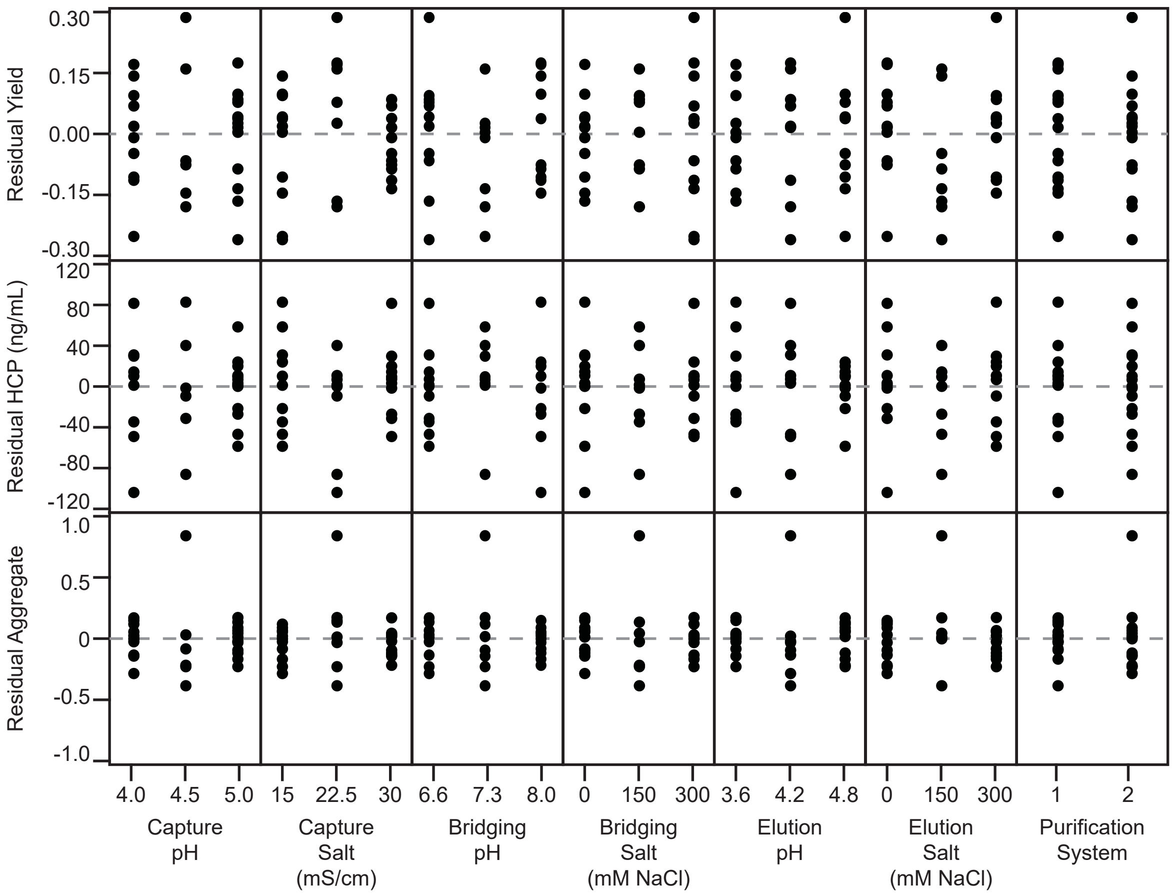

Supplementary Figure 3. Residual plots for the G-CSF process model. Residuals are shown for each response (yield, host-cell protein (HCP), and aggregate) with respect to each input variable (capture pH, capture salt, bridging pH, bridging salt, elution pH, elution salt and purification system).

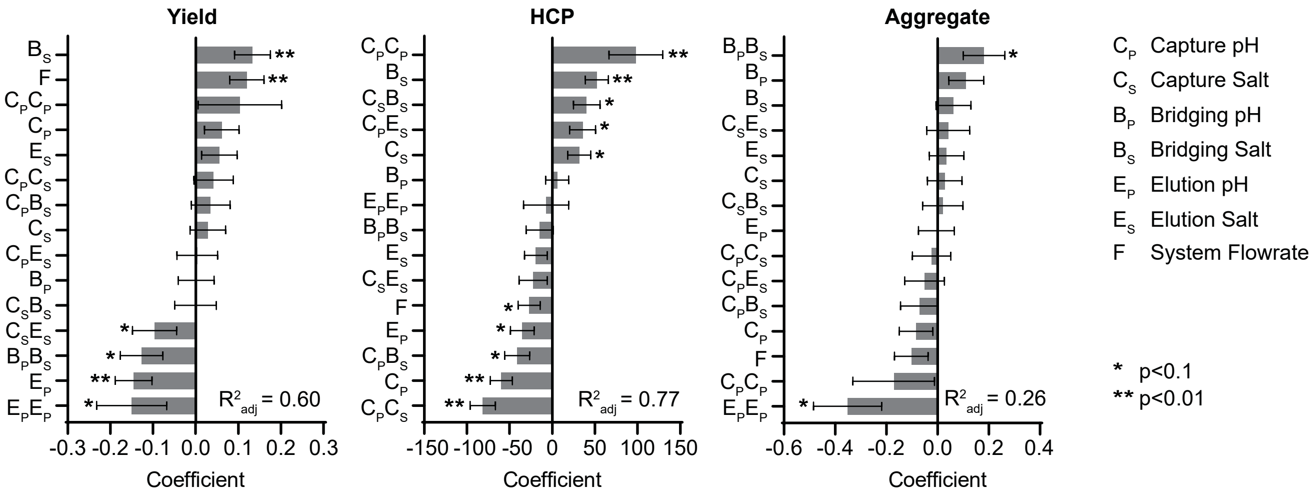

Supplementary Figure 4. Coefficient estimates for each G-CSF process model parameter with respect to each response variable yield, host-cell protein (HCP), or aggregate. Coefficients significantly different than zero based on a two-tailed t-test are marked with an asterisk. Error bars represent standard error. The adjusted R-squared score is shown for each response. C_p – capture buffer pH; C_s capture buffer salt; B_p – bridging buffer pH; B_s – bridging buffer salt; E_p – elution buffer pH; E_s – elution buffer salt; F – system flowrate

Supplementary Table 1. Biophysical characteristics of target molecules

	Molecular Weight (kDa)	pI	GRAVY
**G41**	12.3	6.63	-0.231
**G-CSF**	18.8	5.65	0.209

Supplementary Table 2. Flowrate of different purification systems (DSPs)

Flowpath	Flow Setpoint (mL/min)	Measured Flow (mL/min)	
		DSP1	DSP2
Buffer to waste	1	0.91	0.82
Buffer to column to waste	1	0.90	0.82

Supplementary Table 3. G41 DoE Conditions

Experiment ID	Capture pH	Capture Salt (mS/cm)	Bridging pH	Bridging Salt (mM NaCl)	DSP
1	4.5	15	7.4	300	1
2	5.0	30	6.8	300	1
3	5.0	13	8.0	150	1
4	4.0	30	8.0	300	1
5	5.0	22.5	7.4	0	1
6	4.0	30	8.0	0	1
7	4.5	22.5	6.8	150	1
8	4.0	15	6.8	0	1
9	4.5	22.5	6.8	150	1
10	5.0	15	6.8	150	2
11	4.0	22.5	6.8	300	2
12	5.0	30	8.0	150	2
13	5.0	22.5	8.0	300	2
14	4.5	30	6.8	0	2
15	4.5	15	8.0	0	2
16	4.0	15	8.0	150	2
17	4.0	30	7.4	150	2
18	4.5	22.5	7.4	150	2

Supplementary Table 4. G-CSF DoE Conditions

Experi-mental ID	Capture pH	Capture Salt (mS/cm)	Bridging pH	Bridging Salt (mM NaCl)	Elution pH	Elution Salt (mM NaCl)	DSP
1	5.0	22.5	8.0	300	4.2	0	1
2	5.0	30	7.3	300	4.8	150	1
3	5.0	15	8.0	300	4.8	300	1
4	4.0	22.5	8.0	0	3.6	0	1
5	4.0	15	8.0	0	4.8	300	1
6	4.5	22.5	7.3	150	4.2	150	1
7	4.5	15	8.0	0	3.6	300	1
8	5.0	22.5	6.6	150	4.8	0	1
9	4.5	30	6.6	300	3.6	0	1
10	5.0	30	6.6	150	4.2	300	1
11	4.0	30	6.6	0	4.8	150	1
12	4.0	15	6.6	150	3.6	300	1
13	5.0	30	7.3	0	4.2	0	1
14	4.0	15	7.3	300	4.8	0	1
15	4.0	30	8.0	300	4.2	300	1
16	4.0	15	8.0	300	3.6	150	2
17	5.0	30	8.0	150	3.6	150	2
18	5.0	30	8.0	0	4.8	300	2
19	4.0	15	6.6	0	4.2	0	2
20	5.0	15	6.6	300	4.2	150	2
21	5.0	15	6.6	0	4.8	300	2
22	4.0	30	6.6	300	4.2	0	2
23	4.0	30	7.3	0	3.6	300	2
24	5.0	15	7.3	150	3.6	0	2
25	5.0	22.5	6.6	0	3.6	150	2
26	5.0	22.5	7.3	300	3.6	300	2
27	4.5	22.5	6.6	300	4.8	300	2
28	4.5	30	8.0	150	4.8	0	2
29	5.0	15	8.0	0	4.8	0	2
30	4.5	22.5	7.3	150	4.2	150	2

Supplementary Table 5. G41 DoE Results

Experimental ID	Yield (%)	Host-cell protein (ng/mL)	Host-cell DNA (ng/mL)	Aggregate (%)
Cell Culture Fluid	N/A	195,784 ± 68,548	70,000**	3.91 ± 2.22
1	83.0	284.3	107	<0.05*
2	1.9	176.2	<10*	<0.05*
3	3.4	90.2	<10*	<0.05*
4	86.7	441.1	12	<0.05*
5	57.9	25.3	<10*	<0.05*
6	83.8	187.1	<10*	<0.05*
7	81.8	88.5	<10*	<0.05*
8	99.7	62.9	<10*	<0.05*
9	84.1	79.2	<10*	<0.05*
10	72.7	165.2	<10*	<0.05*
11	79.2	412.1	<10*	<0.05*
12	41.9	61.3	<10*	<0.05*
13	66.2	159.2	<10*	<0.05*
14	53.8	42.8	<10*	<0.05*
15	57.0	67.0	<10*	<0.05*
16	61.1	53.1	<10*	<0.05*
17	73.1	188.9	<10*	<0.05*
18	74.7	139.8	<10*	<0.05*

*Limit of detection

**Typical DNA content of *K. phaffii* supernatant

Supplementary Table 6. G-CSF DoE Results

Experimental ID	Yield (%)	Host-cell protein (ng/mL)	Host-cell DNA (ng/mL)	Aggregate (%)
Cell Culture Fluid	N/A	259,656 ± 21,895	70,000**	N/A
1	86.3	107.3	<10*	0.59
2	51.4	80.0	<10*	<0.05*
3	57.6	142.9	<10*	<0.05*
4	72.7	136.2	<10*	<0.05*
5	37.2	20.7	<10*	<0.05*
6	70.0	112.7	<10*	0.19
7	50.9	163.4	<10*	<0.05*
8	42.7	48.0	<10*	<0.05*
9	79.9	244.1	<10*	<0.05*
10	82.2	56.6	<10*	0.22
11	0.0	N/A	N/A	N/A
12	83.3	72.3	<10*	<0.05*
13	66.1	18.4	<10*	0.19
14	0.0	N/A	N/A	N/A
15	38.3	341.5	<10*	1.14
16	46.7	252.7	<10*	0.70
17	44.7	121.4	<10*	0.34
18	20.1	55.1	<10*	<0.05*
19	0.0	N/A	N/A	N/A
20	43.0	147.2	<10*	0.22
21	0.0	N/A	N/A	N/A
22	66.6	698.3	<10*	0.34
23	17.3	243.9	<10*	0.23
24	23.9	262.1	<10*	<0.05*
25	0.0	N/A	N/A	N/A
26	71.5	227.1	<10*	0.33
27	60.5	116.7	<10*	<0.05*
28	0.0	N/A	N/A	N/A
29	0.0	N/A	N/A	N/A
30	12.2	40.0	<10*	1.62

*Limit of detection

**Typical DNA content of *K. phaffii* supernatant

Supplementary Table 7. Host-cell protein challenge

	Host-cell protein challenge (ng/mL)
This Work	259,656 ± 21,895
Timmick et al.	45,522 ± 154,450
